# *YAP1-MAML2* Fusion as a Diagnostic Biomarker for Metaplastic Thymoma

**DOI:** 10.3389/fonc.2021.692283

**Published:** 2021-07-20

**Authors:** Jikai Zhao, Ruiying Zhao, Chan Xiang, Jinchen Shao, Lianying Guo, Yuchen Han

**Affiliations:** Department of Pathology, Shanghai Chest Hospital, Shanghai Jiao Tong University, Shanghai, China

**Keywords:** metaplastic thymoma, *YAP1-MAML2*, sequencing, FISH, gene rearrangements, biomarker

## Abstract

**Background:**

Metaplastic thymoma is a very rare tumor with only a few case reports documented in literature. Hence, its molecular features have not been well explored.

**Material and Methods:**

Seventeen specimens of metaplastic thymoma were sequenced and retrospectively analyzed by fluorescence *in situ* hybridization (FISH) and immunohistochemistry in the study. In addition, seven cases of micronodular thymoma with lymphoid stroma and nine cases of type A thymoma were also investigated.

**Results:**

Among these metaplastic thymomas, fifteen cases showed classical histological features, and two cases displayed characteristic micronodular-like growth patterns. DNA and RNA based next-generation sequencing identified and confirmed highly recurrent Yes Associated Protein 1 (*YAP1*) - Mastermind Like Transcriptional Coactivator 2 (*MAML2*) translocation (13/17, 76.5%) in metaplastic thymoma but not in micronodular thymoma with lymphoid stroma (0/7, 0%) and type A thymoma (0/9, 0%). In addition, six nonsense mutations were also detected in the metaplastic thymoma. FISH in microdissection specimens indicated that both epithelioid and spindle cell components harbored *YAP1-MAML2* gene rearrangements.

**Conclusions:**

Our study explored the genetic alterations in epithelioid and spindle cell components in metaplastic thymoma. Furthermore, *YAP1-MAML2* gene rearrangements emerged as a potential diagnostic biomarker helpful for distinguishing metaplastic thymoma from type A and micronodular thymoma with lymphoid stroma.

## Introduction

Metaplastic thymoma is an uncommon thymic epithelial malignancy that accounts for less than 1% of all types of thymomas (1). It is a relatively indolent neoplasm that shows biphasic differentiation and comprises of a solid growth epithelial cells along with gradual transiting spindle cells components (2). This terminology for the tumor was introduced in the World Health Organization (WHO) 2004 scheme and continued to the 2020 World Health Organization (WHO) classification for lung and thymus (3, 4).

To our knowledge, only rare cases of metaplastic thymoma have been reported in Asian population (5, 6), whereas about a total of 30 cases have been described separately in the English literatures (7, 8). The genomic alterations driving metaplastic thymoma have not previously been defined in any guidance or classification and hence little is known about its molecular characteristics. Recently, it had been reported that there are high frequency Yes Associated Protein 1 (*YAP1*) - Mastermind Like Transcriptional Coactivator 2 (*MAML2*) fusions in an eight metaplastic thymomas cohort study, and the genetic alterations may be closely related to tumor occurrence or prognosis (9).

In this study, we used a unified RNA and DNA next-generation sequencing (NGS) assay and Fluorescence in Situ Hybridization (FISH) to analyze the molecular characteristics of metaplastic thymoma and compared it with micronodular thymoma with lymphoid stroma as well as type A thymoma.

## Material and Methods

### Patients and Specimens

Seventeen cases of previously diagnosed metaplastic thymoma were collected by screening the archives of the Department of Pathology of Shanghai Chest Hospital from 2010 to 2019. The whole hematoxylin and eosin (H&E) slides of all cases were reviewed by three pathologists with more than ten years of diagnostic experience on thoracic neoplasms. Since type A thymoma and micronodular thymoma with lymphoid stroma (MNTLS) may morphologically overlapped with metaplastic thymoma, we also selected 16 cases, including nine cases of type A thymoma and seven MNTLS. All the samples were surgically resected and their formalin-fixed and paraffin-embedded (FFPE) specimens were used for the study. Clinical information, including age, sex, smoking status, tumor size, and Masaoka-Koga staging of all patients were collected from the medical records. Clinical follow-up data were collected from the consultations of clinicians or obtained from the medical records. The follow-up duration was calculated from the date of surgery to last telephonic follow-up.

This study was approved by the Ethical Committee of Shanghai Chest Hospital of Shanghai Jiao Tong University, Shanghai, China. All patients agreed to participate in the study with all relevant data and written informed consent was obtained from all patients before surgery and subsequent clinical studies. Written informed consents were also obtained from all patients or their legal representatives for the use of surgically resected specimens, as appropriate.

### Immunohistochemical Analysis

A panel of immunohistochemical markers, including cytokeratin (1:400 diluted; clone AE1/AE3; Dako), P63 (1:200 diluted; clone DAK-p63; Dako), TDT (ready-to-use; clone EP266; Dako), CD3 (1:200 diluted; clone SP7), CD20 (1:200 diluted; clone MX003), and CD5 (1:200 diluted; clone 4C7; DAKO), and CD117 (ready-to-use; Polyclonal; Dako) were used for routine and differential diagnosis in the tissue sections of formalin-fixed paraffin-embedded thymoma specimens. 4-5μm representative tissue sections were used and immunohistochemical analysis was performed using the auto-stainer GI100 (DAKO OMNIS; Agilent technologies) and automated stainer (Ventana Benchmark XT; Roche Ventana) following the manufacturer’s instructions. All the samples were stained with H&E in the first and last slides. Immunostained sections were counterstained with hematoxylin. Appropriate positive and negative controls were concurrently run.

### Unified RNA and DNA NGS Assay by PANO-Sequencing Analysis Program

Selected cases were subjected to the parallel amplification numerically optimized (PANO) sequencing assay (10), using a modified on-shelf product (panel #022T, HeliTec Biotechnologies, ShenZhen, China) with designed MAML2 primers spiked in the multiplexed primer pools. This panel can identify all functional fusion events in multiple common genes and genetic variations in all the National Comprehensive Cancer Network (NCCN)-specified biomarkers. To perform this assay, total nucleic acids were first extracted from formalin-fixed paraffin-embedded (FFPE) tissue samples using a PANO-Pure FFPE TNA extraction kit (HeliTec Biotechnologies, ShenZhen, China), and 50 ng of input was used for library construction. This single-tube library construction protocol used DNA for the detection of single nucleotide variants (SNV) as well as insertions and detections (InDels) mutations, RNA for fusion detection, and tiled intronic primers for DNA-based fusion detection when transcripts were unavailable. The reactions were performed in a single tube from extraction to sequencing as a unified library, without experimentally separating DNA or RNA. Sequencing were performed. Raw sequencing data were analyzed using a proprietary PANO-Call ver. 18.12 bioinformatics pipeline for both mutation and fusion calls.

### Fluorescence *In Situ* Hybridization (FISH) Analysis for MAML2 Translocation

Fusion FISH assays were performed on nine metaplastic thymoma cases including six fusion gene-positive cases and three fusion gene-negative cases. 4 μm thick representative tissue sections slides of FFPE tissue sections were used. Epithelioid and spindle cell components were laser-microdissected respectively for analysis using a commercial *MAML2* Break Apart Rearrangement Kit. All slides were deparaffinized, pretreated, hybridized with denatured probes, and incubated overnight. The slides were then washed and stained with 4’,6-diamidino-2-phenylindole (DAPI). The results were visualized using an automatic fluorescence microscope (Zeiss AXI0, Imager.Z2, Germany). The SPEC *MAML2* Dual Color Break Apart Probe is a mixture of two directly labeled probes hybridizing to the 11q21 band. The green fluorochrome direct-labeled probe hybridizes distal to the *MAML2* gene, and the orange fluorochrome direct-labeled probe hybridizes proximal to that gene. At least 200 cells were counted for each section. By calculating the number of positive cells greater than three areas and taking the average value, those cases with more than 10% of tumor cells showing positivity were interpreted as harboring *MAML2* gene rearrangement.

## Results

### Clinical and Pathologic Findings

**Figure 1:**
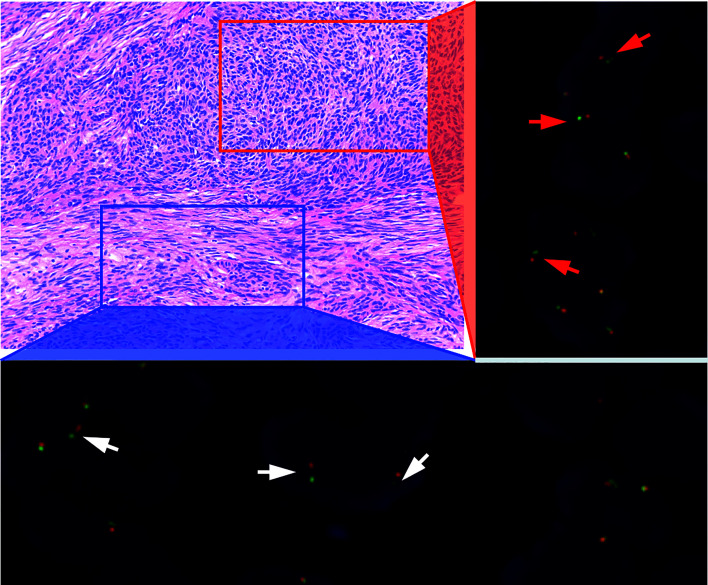
Histological features of metaplastic thymoma. Anastomosing cords or trabeculae of epithelioid cell (red rectangle) and pseudosarcomatous spindle cell components (blue rectangle). H&E staining at 40X magnification. Both epitheloid and spindle cell components harboring *YAP1-MAML2* gene rearrangements are indicated by red and white arrows respectively.

**Figure 2:**
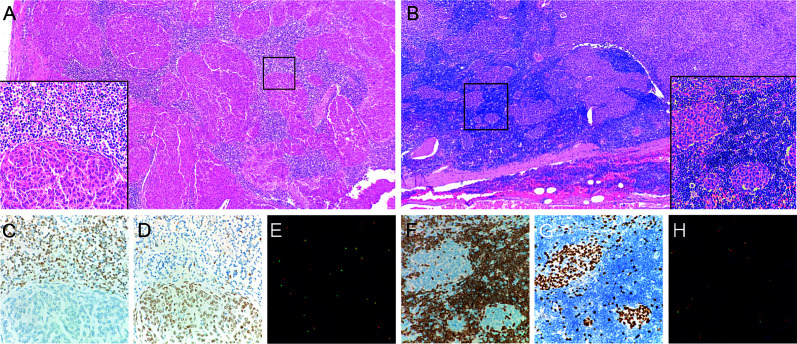
Micronodular-like growth pattern in metaplastic thymoma. **(A)** This growth pattern accounts for 50% of the tumor size in case 5, H&E staining at 4X magnification. The box in the left bottom shows a clear border between epithelioid cell nests and lymphoid stroma, H&E staining at 40X magnification. **(B)** Micronodular-like growth pattern is focally present in case 10, H&E staining at 4X magnification. **(C)** TDT-positive lymphocytes are not present in epithelial cell components of case 5. **(D, G)** P63 outline the epithelial cells. **(F)** TDT-positive lymphocytes are scattered in epithelioid cell nests of case 10. **(E, H)** Micronodular-like growth epithelioid cell nests all display narrow split signals in the assays using *MAML2* dual color break apart probe.

The cohort of 17 metaplastic thymoma patients comprised of nine men and eight women, aged 29-71 years. The tumor size ranged from 1.5 cm to 13.5 cm. Clinical information and prognosis data are presented in **Table 1**. All patients underwent surgical treatment and did not receive postoperative adjuvant therapy. The follow-up duration was eight months to ten years, and no case of tumor recurrence was found until the final follow-up. Clinical information on micronodular thymoma with lymphoid stroma and type A thymoma is listed in **Supplementary Table 1**. Morphologically, most metaplastic thymomas displayed classical histopathology which consisted of solid growth epithelial cell components and mild gradually transitioning spindle cell components in varying proportions (**Figure 1**). Two cases focally or partially showed characteristic micronodular-like growth patterns with abundant lymphoid stroma. However, unlike in MNTLS, the lymphocytic stroma comprised of almost TDT-positive and CD20-negative T lymphocytes with scattered epithelial tumor cells present in the two cases (**Figure 2**). In case 5, the biphasic differentiation of the tumor was not obvious and the epithelial component accounted for more than 90% of the evaluated slides. In case 8, the tumor cells displayed obvious degenerative changes with strange irregular nuclei, intranuclear pseudo-inclusions, but with no proliferate activity. No correlation was observed between morphological differences and fusion gene status. Immunohistochemically, the epithelial islands in all cases were strongly positive for pan-cytokeratin and P63. CD5, CD117 and CD20 expression were negative. TDT- and CD3-positive lymphocytes were only confined to micronodular-like growth areas. The immunohistochemical staining results for tumor cells and lymphoid stroma are listed in **Supplementary Table 2**.

**Table 1:** Clinical information of 17 cases of metaplastic thymoma in this study.

Case no.	Age	Sex	Maximum diameter (cm)	Smoking status	Masaoka-Koga staging	Treatment	Follow-up
1	68	M	7.1	20 packs/years	I	Surgical resection	Alive at 10 years
2	35	M	5.2	non	IIa	Surgical resection	Alive at 7 years
3	65	M	13	200 packs/year	I	Surgical resection	Alive at 4.5 years
4	57	F	2.5	non	IIa	Surgical resection	Alive at 3.5 years
5	29	F	8	non	I	Surgical resection	Alive at 3.5 years
6	69	F	5	non	IIa	Surgical resection	Alive at 3 years
7	57	F	4	non	IIa	Surgical resection	Alive at 2.5 years
8	60	M	3.6	50 packs/year	I	Surgical resection	Alive at 2.5 years
9	36	F	4	non	I	Surgical resection	Alive at 2 years
10	31	M	7.8	non	IIb	Surgical resection	Alive at 2 years
11	45	M	2.2	non	IIa	Surgical resection	Alive at 1.5 years
12	52	M	1.5	non	IIa	Surgical resection	Alive at 1.5 years
13	71	F	5.2	non	IIa	Surgical resection	Alive at 1.5 years
14	55	F	7	non	IIa	Surgical resection	Alive at 1 years
15	52	M	13.5	40 packs/year	I	Surgical resection	Alive at 1 years
16	31	F	3	non	IIa	Surgical resection	Alive at 1 years
17	32	M	10	non	IIa	Surgical resection	Alive at 8 months

### Unified RNA and DNA NGS Assay

Sequencing analysis identified *YAP1*-*MAML2* translocation in 13 out of 17 (76.5%) cases of metaplastic thymomas but not in MNTLs and type A thymomas. Moreover, two metaplastic thymoma cases with micronodular-like growth patterns were also found to harbor this rearrangement. There was no significant correlation between translocation and the proportion of cellular components or growth patterns in metaplastic thymoma. No other fusion events were detected in any of the cases. Intrachromosomal inversion by RNA sequencing identified five products in metaplastic thymoma: 5 *YAP1*_exon 1 fused to 3 *MAML2*_exon 2, 5 *YAP1*_exon 1 fused to 3 *MAML2*_exon 3, 5 *YAP1*_exon 4 fused to 3 *MAML2*_exon 2, 5 *YAP1*_exon 5 fused to 3 *MAML2*_exon 2 and 5 *YAP1*_exon 6 fused to 3 *MAML2*_exon 2. Six nonsense mutations were identified in metaplastic thymoma cases. Four mutations were found in cases with *MAML2* fusions: *ALK* Q1188X in case 1, *CDK4* R5X in case 3, *PTEN* Q110X in case 5, and *CDK4* W179X in case 6. The other two nonsense mutations, *HRAS* Q95X and *BRAF* W476X, were detected in one fusion-negative patient. These nonsense mutations result in the production of a shortened protein, which may be nonfunctional. Breakpoint locations determined by DNA-based sequencing are listed in **Table 2**.

**Table 2:** Unified RNA and DNA NGS Assay and FISH detection of *YAP1-MAML2* fusions in 17 metaplastic thymoma cases.

Case no.	Cellular components			RNA fusions data		DNA sequencing data		FISH result
	Epithelioid (%)	Spindle (%)	micronodular-like (%)	5′ partner	3′ partner	YAP1 breakpoint	MAML2 breakpoint	
1	60	40	None	YAP1_exon1	MAML2_exon2	11_101981900	11_95826684	None detected
2	50	50	None	YAP1_exon4	MAML2_exon2	11_102076805	11_95826684	None detected
3	70	30	None	YAP1_exon4	MAML2_exon2	11_102076805	11_95826684	Positive
4	70	30	None	YAP1_exon1	MAML2_exon2	11_101981900	11_95826684	None detected
5	40	10	50	YAP1_exon4	MAML2_exon2	11_102076805	11_95826684	Positive
6	60	40	None	YAP1_exon1	MAML2_exon2	11_101981869	11_95826682	None detected
7	60	40	None	YAP1_exon4	MAML2_exon2	11_102076805	11_95826684	None detected
8	70	30	None	None	None	None	None	Negative
9	80	20	None	YAP1_exon1	MAML2_exon2	11_101981900	11_95826684	None detected
10	50	40	10	YAP1_exon1	MAML2_exon2	11_101981900	11_95826684	Positive
11	90	10	None	None	None	None	None	None detected
12	60	40	None	YAP1_exon4 YAP1_exon6	MAML2_exon2 MAML2_exon2	11_102076805 11_102080295	11_95826684 11_95826685	Positive
13	70	30	None	None	None	None	None	Negative
14	60	40	None	YAP1_exon1	MAML2_exon2	11_101981900	11_95826684	None detected
15	80	20	None	YAP1_exon4	MAML2_exon2	11_102076805	11_95826684	Positive
				YAP1_exon5	MAML2_exon2	11_102076817	11_95826684	
				YAP1_exon6	MAML2_exon2	11_102080295	11_95826685	
16	80	20	None	YAP1_exon1	MAML2_exon2	11_101981900	11_95826684	Positive
				YAP1_exon1	MAML2_exon3	11_101981901	11_95724889	
17	50	50	None	None	None	None	None	Negative

### Validation of MAML2 Fusions in Metaplastic Thymoma by FISH Verifying

Tumors of nine patients’ harboring fusion conditions including six sequencing fusion-positive cases and three fusion-negative cases were detected by FISH method in microdissection slides. The results revealed that both epithelioid and spindle cell components harboring *YAP1*-*MAML2* fusions in positive cases, including two tumors with micronodular-like growth pattern (**Figures 1** and **2**).

## Discussion

Our molecular findings confirmed a higher frequency of *YAP1*-*MAML2* gene rearrangements in metaplastic thymomas. In addition, six nonsense mutations were identified in these cases, including *ALK* Q1188X, *CDK4* R5X, *PTEN* Q110X, and *CDK4* W179X in four fusion gene-positive cases, and *HRAS* Q95X along with *BRAF* W476X in one fusion gene-negative case. The MAML2 gene rearrangements were also studied in micronodular thymoma with lymphoid stroma and conventional type A thymoma, but no rearrangements were found in these types. Furthermore, we identified a micronodular-like growth pattern in two cases of metaplastic thymoma.

Thymic epithelial tumors are traditionally classified into four groups: spindle cell thymoma, predominantly lymphocytic thymoma, mixed lymphocytic and epithelial thymoma, and predominantly epithelial thymoma, according to the lymphocyte-to-epithelial cell ratio and the shape of epithelial cells (11). Taking into account the prognosis and molecular alterations, the 2004^th^ and 2015^th^ WHO classification of tumors of the thymus modified and revised the nomenclature (4, 12). Occasionally, in addition to spindle cell morphology, type A thymoma demonstrates various histological patterns including rosettes with or without central lumens, papillary projections in cystic spaces, or meningioma-like wholes, and may mimic a biphasic growth pattern that is often devoid of immature T cells. In our cohort, we also identified cases of metaplastic thymoma with abundant lymphoid stroma. Two patients had abundant lymphoid stroma mimicking the growth pattern of micronodular thymoma, and one patient had symptoms of slight blepharoptosis. Unlike MNTLS in which most of the lymphocytes, were mature B lymphocytes, the lymphoid cells of the stroma in these two metaplastic thymoma cases were TDT-positive T lymphocytes, and these lymphocytes would or would not be present in the epithelial nests. However, scattered tumor epithelial cells could always be found in the lymphoid stroma, reminiscent of type B1 thymoma. Miki et al. have suggested a relationship between metaplastic thymoma and type AB thymoma (13). Therefore, it is necessary to distinguish metaplastic thymoma from type A and B in clinical practice, as it is quite challenging in individual cases by only morphological evaluation.

The epithelial-mesenchymal transition seems to be involved in the transitional differentiation of epithelioid and spindle cell components. Moreover, the histogenesis and relationship between metaplastic thymoma and other thymic epithelial neoplasms remains unknown (14). Sarcomatoid transformation was not found in metaplastic thymoma cases that were included in our study as per the follow-up data available, and this phenomenon has been reported in very few studies (15, 16).

There may be morphological or even genetic alterations that overlap between metaplastic thymoma, type A/AB and MNTLS, but chromosomal abnormalities by comparative genomic hybridization and previous molecular studies have shown no evidence to support these conjectures (17). Marina et al. have reported a recurrent genetic signature of *YAP1-MAML2* fusion in metaplastic thymoma for the first time and described two distinct *YAP1-MAML2* transcription products (9). Our results confirm a high frequency of *YAP1-MAML2* fusions in metaplastic thymoma cases, suggesting that it may be a common pathogenetic mechanism in the development of metaplastic thymomas. Although there was a certain degree of morphological overlap among metaplastic thymoma, MNTLS or even type A/AB thymoma, it basically did not affect the accurate pathological typing. In addition, sequencing results have also suggested that the histogenesis mechanism of metaplastic thymoma may be different from MNTLS and type A/AB thymoma. In a recent study, Lucas et al. have described a recurrent gene rearrangement of *KMT2A-MAML2* in type B2 and B3 thymomas (18). Fusion with different gene partners suggests that *MAML2* gene rearrangement may be a potential biomarker for the morphological classification of thymomas.

Recurrent *YAP1-MAML2* fusions have been reported in poroma and porocarcinomas, pediatric *NF2*‐wildtype meningioma, composite and retiform hemangioendothelioma, glioblastoma, nasopharyngeal carcinoma and ovarian cancer cell lines (19–24). However, its function is poorly understood. *YAP1* and *MAML2* are on the opposite strands of adjacent genetic loci on the p arm chromosome 11, and the co-expression of *YAP1-MAML2* fusion transcripts is thought to be the consequence of intrachromosomal inversions. Gabriele et al. have validated the fusion and found that *YAP1-MAML2* fusion is associated with increased *YAP1* signaling and further driving the Hippo signaling cascade in *YAP1-MAML2* tumors (25). Previous studies on multiple solid tumors suggest that *YAP1* aberrant activation is related to poor prognosis, chemoresistance, and resistance to cell death (26, 27). However, the biologic behavior of metaplastic thymoma appears indolent, and the function of *YAP1-MAML2* fusion in metaplastic thymoma needs to be further explored.

In conclusion, the present study further confirms and emphasizes the fundamental genetic alterations of *YAP1-MAML2* gene rearrangements in metaplastic thymomas. Moreover, we identified two cases of metaplastic thymoma with a micronodular growth pattern. Our findings also indicate that the *YAP1-MAML2* gene rearrangements may be a useful diagnostic biomarker for distinguishing metaplastic thymoma from type A thymoma and micronodular thymoma with lymphoid stroma.

## Data Availability Statement

The datasets presented in this study can be found in online repositories. The names of the repository/repositories and accession number(s) can be found in the article/**Supplementary Material**.

## Ethics Statement

The studies involving human participants were reviewed and approved by Ethical Committee of Shanghai Chest Hospital of Shanghai Jiao Tong University. The patients/participants provided their written informed consent to participate in this study.

## Author Contributions

YH designed the study. RZ and LG performed next-generation sequencing and analyzed the data of FISH. JZ, JS, and YH performed morphological evaluation and LG completed immunohistochemical staining. RZ and CX analyzed and interpreted the sequencing data. JZ wrote the main manuscript. YH and RZ participated in the writing part of the discussion. JZ and RZ contributed equally to this work. All authors contributed to the article and approved the submitted version.

## Funding

The study was funded by Nurture Projects for Basic Research of Shanghai Chest Hospital (Grant number: 2020YNJCM13).

## Conflict of Interest

The authors declare that the research was conducted in the absence of any commercial or financial relationships that could be construed as a potential conflict of interest.

## Supplementary Material

Supplementary Table 1 Clinical information of micronodular thymoma with lymphoid stroma (MNTLS) and type A thymoma cases used in this study.

Supplementary Table 2 The immunohistochemical staining results for tumor cells and lymphocytes components in metaplastic thymoma.

## Abbreviations

FISH, fluorescence *in situ* hybridization; *YAP1*, Yes Associated Protein 1; *MAML2*, Mastermind Like Transcriptional Coactivator 2; WHO, World Health Organization; NGS, next generation sequencing; H&E, hematoxylin and eosin; MNTLS, micronodular thymoma with lymphoid stroma.
