# Supplementary material for: YAP1-MAML2 Fusion as a Diagnostic Biomarker for Metaplastic Thymoma
Source: Front Oncol. 2021 Jul 20;11:692283. doi: 10.3389/fonc.2021.692283 (PMC8329546; doi:10.3389/fonc.2021.692283)
Supplement: Supplementary Table 1 — Clinical information of micronodular thymoma with lymphoid stroma (MNTLS) and type A thymoma cases used in this study. [file Table_1.docx]

Supplementary Table 1. Clinical information of micronodular thymoma with lymphoid stroma (MNTLS) and type A thymoma cases used in this study.

| Case no. | Age | Sex | Maximum  diameter (cm) | Smoking status | Masaoka-Koga  staging | Histology | Treatment | Follow-up |
| --- | --- | --- | --- | --- | --- | --- | --- | --- |
| 1 | 53 | M | 2 | non | IIb | MNTLS | Surgical resection | Alive at 3 years |
| 2 | 65 | M | 7 | 50 packs/year | IIb | MNTLS | Surgical resection | Alive at 2.5 years |
| 3 | 80 | M | 6.5 | 90 packs/year | IIb | MNTLS | Surgical resection | Died of other disease at 2.5 years |
| 4 | 64 | F | 4 | non | IIa | MNTLS | Surgical resection | Alive at 2.5 years |
| 5 | 64 | M | 5 | non | IIb | MNTLS | Surgical resection | Alive at 2 years |
| 6 | 67 | F | 2 | non | IIb | MNTLS | Surgical resection | Alive at 2 years |
| 7 | 62 | F | 4 | non | IIa | MNTLS | Surgical resection | Alive at 6 years |
| 8 | 65 | F | 8.5 | non | Ⅲ | Type A | Chemotherapy after surgical resection | Lung metastasis, 4.5 years after resection |
| 9 | 62 | M | 4 | 50 packs/year | IIb | Type A | Surgical resection | Alive at 3 years |
| 10 | 77 | F | 17 | non | I | Type A | Surgical resection | Lost follow-up |
| 11 | 77 | F | 3.6 | non | IIa | Type A | Surgical resection | Alive at 3 years |
| 12 | 71 | M | 13 | 50 packs/year | IIa | Type A | Surgical resection | Died of heart disease at 2 years |
| 13 | 79 | F | 5.5 | non | IIa | Type A | Surgical resection | Lost follow-up |
| 14 | 47 | M | 5.5 | non | Ⅳb | Type A | Surgical resection and chemotherapy | Alive at 2 years |
| 15 | 54 | F | 1.7 | non | IIa | Type A | Surgical resection | Alive at 2 years |
| 16 | 65 | F | 15 | non | IIa | Type A | Surgical resection after radiotherapy | Lung metastasis, 3 years after resection |
