# Supplementary material for: YAP1-MAML2 Fusion as a Diagnostic Biomarker for Metaplastic Thymoma
Source: Front Oncol. 2021 Jul 20;11:692283. doi: 10.3389/fonc.2021.692283 (PMC8329546; doi:10.3389/fonc.2021.692283)
Supplement: Supplementary Table 2 — The immunohistochemical staining results for tumor cells and lymphocytes components in metaplastic thymoma. [file Table_2.docx]

Supplementary Table 2. The immunohistochemical staining results for tumor cells and lymphocytes components in metaplastic thymoma.

| Case no. | CK | |  | P63 | |  | TDT | |  | CD3/CD5 | |  | CD20/CD117 | |
| --- | --- | --- | --- | --- | --- | --- | --- | --- | --- | --- | --- | --- | --- | --- |
|  | Tumor cells | lymphocytes |  | Tumor cells | lymphocytes |  | Tumor cells | lymphocytes |  | Tumor cells | lymphocytes |  | Tumor cells | lymphocytes |
| 1 | Positive | Negative |  | Positive | Negative |  | Negative | Scattered |  | Negative | Scattered |  | Negative | Negative |
| 2 | Positive | Negative |  | Positive | Negative |  | Negative | Negative |  | Negative | Negative |  | Negative | Negative |
| 3 | Partial | Negative |  | Partial | Negative |  | Negative | Negative |  | Negative | Negative |  | Negative | Negative |
| 4 | Positive | Negative |  | Positive | Negative |  | Negative | Negative |  | Negative | Negative |  | Negative | Negative |
| 5 | Partial | Negative |  | Partial | Negative |  | Negative | Positive |  | Negative | Positive |  | Negative | Negative |
| 6 | Positive | Negative |  | Positive | Negative |  | Negative | Negative |  | Negative | Negative |  | Negative | Negative |
| 7 | Positive | Negative |  | Positive | Negative |  | Negative | Negative |  | Negative | Negative |  | Negative | Negative |
| 8 | Positive | Negative |  | Positive | Negative |  | Negative | Negative |  | Negative | Focal |  | Negative | Negative |
| 9 | Partial | Negative |  | Partial | Negative |  | Negative | Negative |  | Negative | Negative |  | Negative | Negative |
| 10 | Positive | Negative |  | Positive | Negative |  | Negative | Positive |  | Negative | Positive |  | Negative | Negative |
| 11 | Positive | Negative |  | Positive | Negative |  | Negative | Negative |  | Negative | Negative |  | Negative | Negative |
| 12 | Positive | Negative |  | Positive | Negative |  | Negative | Negative |  | Negative | Negative |  | Negative | Negative |
| 13 | Partial | Negative |  | Partial | Negative |  | Negative | Negative |  | Negative | Focal |  | Negative | Negative |
| 14 | Positive | Negative |  | Positive | Negative |  | Negative | Negative |  | Negative | Negative |  | Negative | Negative |
| 15 | Positive | Negative |  | Positive | Negative |  | Negative | Scattered |  | Negative | Scattered |  | Negative | Negative |
| 16 | Positive | Negative |  | Positive | Negative |  | Negative | Negative |  | Negative | Negative |  | Negative | Negative |
| 17 | Positive | Negative |  | Partial | Negative |  | Negative | Negative |  | Negative | Scattered |  | Negative | Negative |
